# Recent Advances in Biopolymer-Based Dye Removal Technologies

**DOI:** 10.3390/molecules26154697

**Published:** 2021-08-03

**Authors:** Rohan S. Dassanayake, Sanjit Acharya, Noureddine Abidi

**Affiliations:** 1 Department of Biosystems Technology, Faculty of Technology, University of Sri Jayewardenepura, Nugegoda 10250, Sri Lanka; rdassanayake@sjp.ac.lk; 2 Fiber and Biopolymer Research Institute, Texas Tech University, Lubbock, TX 79409, USA; sanjit.acharya@ttu.edu

**Keywords:** dye removal, biopolymers, adsorption, advanced oxidation processes, membrane filtration, cellulose, chitin, chitosan

## Abstract

Synthetic dyes have become an integral part of many industries such as textiles, tannin and even food and pharmaceuticals. Industrial dye effluents from various dye utilizing industries are considered harmful to the environment and human health due to their intense color, toxicity and carcinogenic nature. To mitigate environmental and public health related issues, different techniques of dye remediation have been widely investigated. However, efficient and cost-effective methods of dye removal have not been fully established yet. This paper highlights and presents a review of recent literature on the utilization of the most widely available biopolymers, specifically, cellulose, chitin and chitosan-based products for dye removal. The focus has been limited to the three most widely explored technologies: adsorption, advanced oxidation processes and membrane filtration. Due to their high efficiency in dye removal coupled with environmental benignity, scalability, low cost and non-toxicity, biopolymer-based dye removal technologies have the potential to become sustainable alternatives for the remediation of industrial dye effluents as well as contaminated water bodies.

## 1. Introduction

Imparting color to consumer goods such as textiles is customary practice in many industries [1]. Primarily, it is done to make the products aesthetically pleasant, thereby making them more appealing to the consumers. Indeed, human eyes are incredibly adept at detecting even miniscule color differences. Color could be one of the important determining factors for the consumers in making purchasing decisions, e.g., a textile garment [1,2]. In fact, humans have always been intrigued by colors. For instance, the use of colored garments could be traced back to prehistoric times as early as 3500 BCE in the human civilization [3,4]. Prior to the serendipitous discovery of the first synthetic dye, mauve, by German chemist William Henry Perkins in 1856 [5], dyes and pigments used in coloring of consumer products were derived from natural sources, including vegetables, flowers, woods and insects [3,6]. However, due to numerous desirable attributes such as large-scale production ability, water solubility, versatility in colors and high fastness as compared to natural dyes and pigments, present day industry almost solely depends on synthetic dyes. Moreover, synthetic organic dyes have become indispensable to numerous industries such as textiles, paper, plastics, and even in food and pharmaceuticals [7]. According to several references, currently more than 100,000 different chemicals are synthesized as dyes, and their estimated total annual global production varies from 700,000 to 800,000 tons [8,9,10]. Even though dyes are utilized in numerous industries, the textile industry is the largest consumer of the produced dyes. Unfortunately, often, the efficiency of the industrial textile dyeing process is substantially low. Due to poor exhaustion of dyes onto the textile fibers from the dyebath and poor fixation of dye molecules onto the textile substrate, a considerable amount of dyes is released in the effluent. This makes the textile industry the leading generator of dye effluent. Loss as high as 15% of the total dyestuff is reported in the textile industries and is later released in the effluents [11]. Besides the textile industry, other major sources of dye effluents are the paper and pulp industry, tannery and paint industry and dye manufacturing industry [8].

Dye effluents, popularly also known as dye wastewater, especially from textile dye houses, not only contain dyes but are also rich in other chemicals such as high amount of salts, alkali, acids, surfactants and metals, which are added to the dye mixture to enhance exhaustion and fixation of dyes.

Owing to their intense visibility, their recalcitrance to biodegradation, and potential toxicity and carcinogenicity of many industrially important synthetic dyes, the inevitable release of dyes in the effluents, even in small concentration, from various dye-utilizing industries including textiles, tannin and leather, has severe environmental and health impacts [10,11]. The presence or discharge of dye effluents to the water bodies even in relatively small quantities makes the receiving water virtually unusable for humans and animal consumption, such as drinking, cooking, bathing and washing [12].

Pollution resulting from the disposal of dye effluents is both an environmental and health concern of high importance. Therefore, it is imperative to find efficient dye remediation techniques, which could be employed both at the polluted site and site of origin, including dye houses. However, successful removal of the colored compounds is considered one of the most challenging tasks encountered by wastewater treatment plants (WWTPs) of textile industries [13]. The reason being the synthetic dyes are complex molecules and are usually purposely designed to make them durable by resisting bio, photo and oxidative degradation [14,15,16,17]. Therefore, they remain in the environment for a considerable time. For instance, the hydrolyzed Reactive Blue 19, a commercial textile dye, has a half-life of approximately 46 years at neutral pH and ambient temperature (25 °C) [18]. Another critical factor, which exacerbates the situation during remediation of dyes from wastewater or other environments, is secondary pollution. The breakdown of dye molecules present in the wastewater often results in the generation of more nefarious by-products, including chlorinated organics, phenols and heavy metals [19,20].

Furthermore, the cost associated with dye removal or remediation technologies is also a key factor. Ideally, a dye removal method should be capable of rapidly removing large quantities of dyes from wastewater without generating secondary pollution and cheaper. Technologies for dye removal can be divided into three categories: biological (fungal decolorization, microbial decolorization, adsorption by living or dead microbial biomass, and anaerobic bioremediation), chemical (advanced oxidation processes (AOPs), electrochemical process, and coagulation or flocculation) and physical methods (adsorption, membrane filtration, ion exchange, electro-kinetic coagulation).

Techniques including adsorption, biodegradation, oxidative degradation, electrochemical destruction, ozonation, photochemical and UV assisted degradation have been commonly reported in the literature; however, an efficient and sustainable approach for removal/remediation of dyes from effluents is yet to be established [8]. Since effluents from a wide range of textile and dye manufacturing industries are generated and variations in their composition based on the types of the manufacturing process and products, no specific treatment or remediation method can be employed at all times and places. Instead, a combinatory approach comprised of physical, chemical, and biological methods is more appropriate [21]. Since cost is the significant factor for any process/technology to find eventual adoption in industries, an efficient and widely available cheaper substitutes-based methods of dye removal/remediation would be highly desirable to address cost issues [22]. There is numerous literature available for the usage of a wide range of materials, including natural and synthetic materials for dye removal technologies. However, this review highlights the applications of biopolymers in three different dye removal technologies: adsorption, AOPs, and membrane filtration. Adsorption and membrane filtration are physical methods of dye removal. The dye removal using these methods are commonly achieved by mass transfer mechanism where solutes (dye molecules) are transferred from their solution or gaseous surroundings and accumulated on the substrate either due to favorable chemical or physical interaction between dye molecules and a substrate (e.g., common adsorption methods) [8,23]. The accumulation of dyes on a substrate can also be achieved by substrate acting as a selective barrier of free movement of dye molecules while solvent can pass through (e.g., membrane filtration) [24]. The physical methods, especially adsorption, are the most versatile and commonly used dye removal methods. AOPs, one of the conventional chemical dye removal methods, involve the degradation of dye molecules by strong oxidants, such as radical and peroxides [25,26].

## 2. Different Types of Dyes

**Figure 1:**
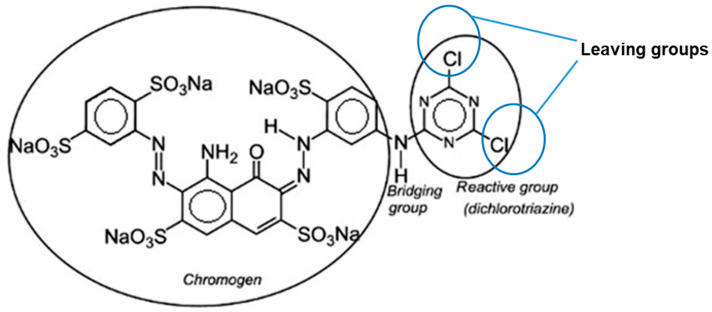
Chemical structure of CI Reactive Blue 109, a dichlorotriazines type reactive dye. (Adapted with permission from reference [29], Copyright 2017 Elsevier).

Dyes are colorful compounds designed to impart color to the substrates. They are anchored on substrates such as yarns, fabrics, and plastics. They are also used to color liquids such as gasoline by mixing. Therefore, the common feature of dyes is their ability to absorb or emit light in the visible region (400–700 nm) to produce color [11]. In addition to the chromogene–chromophoric system, the moiety responsible for producing color, a dye may contain other chemical groups such as bridging groups (auxochromes) and reactive solubilizing groups (Figure 1). Based on the intended application, anchoring methods such as physical adsorption or covalent fixation, and hue of the color, the synthetic dyes can have diverse chemical structures. Typically, the chromogene is an aromatic structure, usually comprised of benzene, naphthalene, or anthracene rings. These rings carry binding chromophores containing double conjugated links having delocalized electrons to form conjugated systems. Chromophores are usually the azo group (–N=N–), methine group (–CH=), ethylene group (=C=C=), carbon-sulfur (=C=S; ≡CS–S–C≡), carbonyl group (=C=O), nitro (-NO_2_; –NO–OH), nitroso (–N=O; =N–OH), carbon-nitrogen (=C=NH; –CH=N–) or chinoid groups. The ionizable groups, such as –COOH (carboxyl), –NH_2_ (amino), –SO_3_H (sulfonate) and –OH (hydroxyl) are the common auxochromes and are responsible for the binding capacity of the dye molecules onto the textile materials [4,27]. Reactive dyes, one of the most popular classes of textile dyes, consist of functional groups that can be covalently linked to the substrate, such as cellulosic fibers. The reactive functional groups may contain labile fluorine, chlorine, methyl sulfone, or nicotinyl leaving groups (see Figure 1). The reaction between the substrate and dyes occurs via nucleophilic substitution at leaving groups (–C–Cl), activated by the adjacent nitrogen present in the triazine ring [28], see Figure 1.

**Figure 2:**
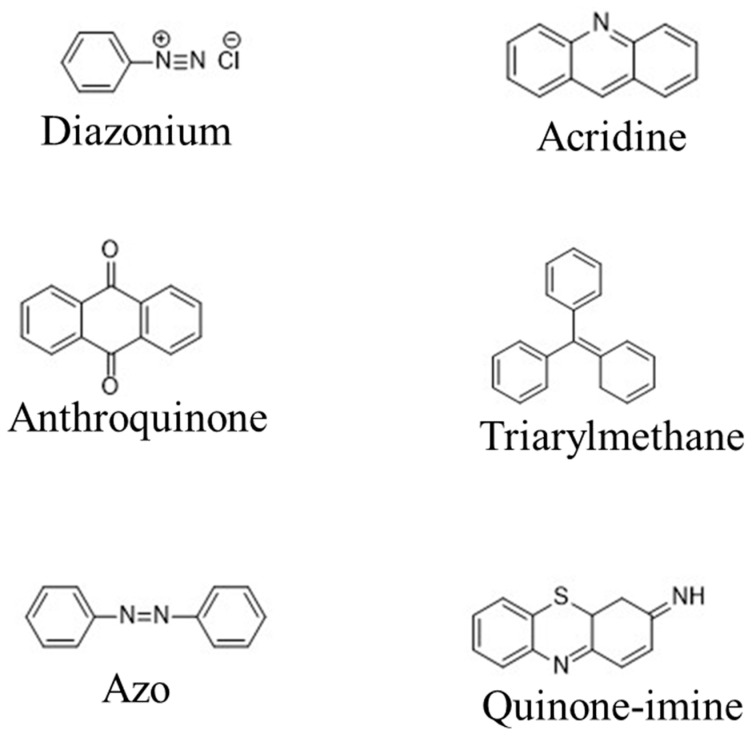

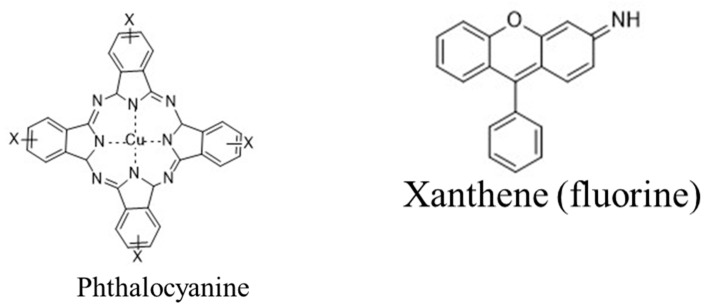
Molecular structure of some of the diverse classes of textile dyes. (Adapted with permission from reference [7], Copyright 2012 Elsevier).

Synthetic dyes can be catalogued based on different parameters, including the molecular structure, methods of application, solubility, ionic and basic or acidic nature [7,8]. Classification of dyes based on the chromophore groups is considered a more systematic approach [7]. Azo dyes, acridine dyes, arylmethane dyes, anthraquinone dyes, nitro dyes, xanthenes dyes and quinine–amine dyes (Figure 2) are some of the important classes of dyes based on their chromophore groups [7]. They make the largest group of synthetic organic chemicals with a 70% share of all produced dyes worldwide [7,30]. The main characteristic of azo dyes is their nitrogen-to-nitrogen double bonds (–N=N–) that are linked to at least one aromatic group, either benzene or naphthalene. The azo dyes can be further subdivided into monoazo, diazo and triazo types. Some of the typical azo dyes are Acid Orange 7, Methylene Orange, Acid Orange 20, Orange II, Methyl Red, Reactive Red 2, Reactive Orange 16, Reactive Black 5, Congo Red, Solvent Red 19, Direct Blue 160, Basic Yellow 15, Basic Blue 41, Disperse Orange 1, Disperse Red 1, Amido Black, Reactive Orange 16, and Amaranth [7,31]. Despite notoriety because of their toxicity and other hazardous effects, azo dyes are the most widely used dye type across many different industries, including textiles, leathers, papers and even in food and cosmetics [7,24,32]. For example, Acid Orange 7 is used in paper, textile and leather industries [33], Solvent Red 19 is usually added in gasoline (diesel) [31], and Amaranth is mainly used to color food and cosmetics industries [32]. Different types of dyes based on other classification methods, their solubility, and major applications are presented in Table 1.

## 3. Biopolymer-based Dye Removal Technologies

With the rapid increase in the use of industrial dyes over the last few decades, different technologies useful for the removal of dyes from aqueous solutions have been investigated. More than a thousand research articles have been reported in the literature on dye removal technologies indicating the high demand for new techniques. The recent advancements in dye removal technologies include adsorption, advanced oxidation processes, chemical coagulation-flocculation, electrochemical treatment, membrane filtration, biological treatment, reverse micelle extraction and other techniques [34]. Albeit efforts have been made to develop various dye removal technologies, there are many challenges associated with them. The major setbacks of commonly used dye removal technologies include high production cost, high labor involvement, and complexity. Many of those technologies use petroleum-based materials, raising concerns over their environmental impact as they are derived from non-renewable and non-biodegradable sources. Furthermore, chemically synthesized materials are practically inapplicable due to multi-step synthesis processes and the use of many toxic chemicals, including organic solvents. Interestingly, natural polymers or biopolymers including cellulose, chitin and chitosan have recently been considered for dye removal technologies due to their relative abundance, low cost, tunable properties such as surface area, pore size, pore volume, ease of handling and environmental benignity. Biopolymers are biodegradable and possess an added advantage over synthetic materials, exhibiting no adverse effect on the environment or living beings. Biopolymers are biocompatible and derive from renewable sources, making them sustainable alternatives for petroleum-based or chemically synthesized materials. Due to the presence of functional groups including hydroxyl (–OH), amine (–NH_2_), carboxyl (–COO^−^) and amide (–NHCOCH_3_), surface functionalization of biopolymers is relatively easy without influencing the biological and physicochemical properties of the surface. Surface-modified biopolymers show enhanced compatibility, rigidity, flexibility, thermal and chemical stability and fast response [35]. Biopolymer-based materials can be fabricated into a diverse range of structures, including spherical, films, cylindrical, bead, fibers, scaffolds, blocks, micro- and nanoparticles, and micro- and nanofibers. However, current applications of biopolymers are also associated with several setbacks, including microbial contamination, variations in the properties as a result of the source of the material, presence of other materials and impurities requiring an initial purification process. This review summarizes current developments of the three major biopolymer-based dye removal technologies: adsorption, AOPs and membrane filtration and their major advantages and limitations.

### 3.1. Adsorption

Adsorption is a surface process that leads to the accumulation of a substance (a molecule, an ion or atom) on the solid surface from its gaseous or liquid surroundings. The substance that accumulates on the surface of the solid is called the adsorbate, and the solid surface on which the adsorption process occurs is called the adsorbent [34]. Depending on how the adsorbates are adsorbed onto the adsorbent surface, the adsorption process can be classified into physisorption (or physical adsorption) and chemisorption (or chemical adsorption) [36,37]. Physisorption occurs via weak intermolecular interactions, including electrostatic, π-π, hydrophobic, dipole–dipole, Van der Waals interactions, and hydrogen bonding between the adsorbate and adsorbent [36,37,38]. Physisorption is a reversible process (also known as desorption) and can result in monolayer or multilayer adsorption [39]. On the contrary, chemisorption is an irreversible process that occurs via strong chemical interactions between the adsorbate and the adsorbent [36,37,38]. Examples of such strong interactions include covalent or ionic bonding [37].

The adsorption process is described at the equilibrium using adsorption isotherms that quantify the amount of the substance adsorbed per unit mass of adsorbent as a function of the equilibrium concentration of the adsorbate [40]. Commonly used adsorption isotherms are the Langmuir and Freundlich isotherm models [41]. Other adsorption isotherms include Tempkin, Dubinin–Radushkevich, Flory–Huggins, Hill, and Redlich–Peterson, Toth, Radke–Prausnitz, Koble–Corrigan, Sips, Fritz–Schluender, Jovanovic, Brunauer–Emmett–Teller, MacMillan–Teller, Vieth-Sladek, Harkins–Jura, Halsey, Frenkel–Halsey–Hill, Henderson, Valenzuela-Myers, Baudu and Elovich [42].

Adsorption technologies have widely been employed for dye removal due to their relatively simple design, safe handling, high treatment efficiency, cost-effectiveness and sludge-free cleaning operation [43,44]. Moreover, the adsorption process does not produce toxic substances, and adsorbents can be regenerated for multiple cycles [34]. Major factors that influence the adsorption efficiency are the initial adsorbate concentration, pH, temperature, adsorbent particle size, porosity and surface area, adsorbate and adsorbent interaction, adsorbent to adsorbate ratio and contact time [15,34,37]. Adsorbents such as activated carbon (AC), clays, zeolites, alumina, silica gel, composites, biomasses and other types of biological and polymeric adsorbents have been tested for the removal of dyes from aqueous solutions [45,46,47,48,49,50,51,52,53,54,55,56]. Among all those adsorbents, this review only aims at the latest literature on the three main biopolymer-based adsorbents derived from cellulose, chitin and chitosan reported for the removal of various dyes from aqueous solutions and industrial effluents. There is a vast body of literature reporting the removal of industrial dyes using different types of biopolymer-based adsorbents. However, the focus of this section is to summarize some of the most widely tested adsorbent types. Biopolymer-based adsorbents including activated carbon (AC), aerogels, hydrogels, microspheres, beads, sponges, metal-oxide and polymeric composites, and nanofibrils have been broadly applied for dye removal applications.

Tony reported chemically activated cellulose-based adsorbent from sugarcane bagasse for treating industrial dye, Procion Blue MX-7RX (reactive blue 161) [57]. Wang and coworkers examined the dye adsorption capacities of activated carbon prepared from chemical activation and pyrolysis of sodium carboxymethyl cellulose (CMC) against three dye molecules: methyl violet (MV), Allura red (AR) and Congo red (CR) [58]. The maximum adsorption capacities reported were 1351.4 (pH 6, 25 °C), 223.2 (pH 7, 45 °C) and 1779.5 (pH 7, 25 °C) mg/g for MV, AR, and CR, respectively. Yu et al. fabricated three-dimensional (3D) nitrogen-doped activated carbon aerogels derived from sodium carboxymethyl cellulose and tested the removal of four different organic dyes [59]. They reported adsorption capacities of 238.2, 230.4, 85.2 and 73.3 mg/g for malachite green (MG), methylene blue (MB), Congo red (CR) and Remazol turquoise blue G-133 (TRB G133), respectively. Wang and coworkers prepared cellulose-based hydrophobic carbon aerogels and showed the removal of various industrial dyes, including methyl blue (MB), alizarin yellow (AY), methyl orange (MO), calcein, amido black 10B, malachite green (MG) and rose Bengal (RB) [60]. The adsorption capacities of those dyes ranged from 195 to 1947 mg/g. Harada et al. prepared activated carbon-containing 3D-porous cellulose beads (ACPBs) and used them for the removal of toluidine blue (TB) dye as a model adsorbate [61]. The maximum adsorption capacity of ACPBs for TB was 123.5 mg/g. The reusability studies of ACPBs showed an adsorption efficiency of around 85% after three consecutive cycles. Wan and coworkers reported the adsorption of Acid Orange 7 dye onto a 3D porous cellulose-based microsphere material (CMs4) functionalized with ionic liquids [62]. The adsorption capacity of the CMs4 sample was 218.6 mg/g. The desorption and reusability studies showed that the adsorption capacity was about 97.2% after three regeneration cycles [53]. Jiang et al. synthesized ultra-light cellulose nanofibrils aerogels and investigated the removal of the cationic malachite green (MG) dye from aqueous media [63]. Cellulose- Fe_3_O_4_ composite was prepared and tested for the removal of Congo red by Srasri et al. [64]. Li and coworkers examined the adsorption capacity of carbonylated cellulose fiber/microfibrillated cellulose (MCMFCs) composite beads using methylene blue (MB). The MCMFCs had a maximum adsorption capacity of MB at 303 mg/g [65]. Porous cellulose nanocrystal aerogels fabricated by cross-linking with poly (methyl vinyl ether-co-maleic acid) (PMVEMA) and poly(ethylene glycol) (PEG) were tested for the removal of methylene blue (MB) by Liang et al. [66]. They reported a maximum adsorption capacity of 116.2 mg/g for the CNC-derived polymeric adsorbent (CNC/PMVEMA/PEG) with a similar performance over five adsorption/desorption cycles.

**Scheme 1:**
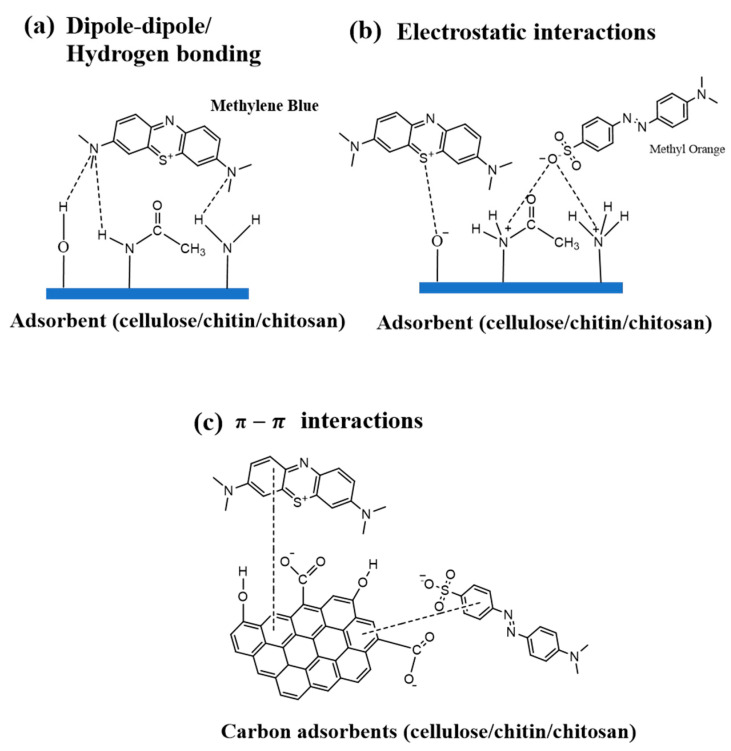
Schematic representation of the three major adsorption mechanisms of dye molecules (MB and MO) by cellulose, chitin, chitosan and their carbon materials: (**a**) H-bonding/dipole–dipole, (**b**) electrostatic and (**c**) π-π interactions.

Several review articles have been recently published on dye removal and other applications of chitin and chitosan and their derivatives. Peter et al. reviewed the unmodified chitin and chitosan along with their derivatives in dye adsorption applications [67]. Kumar and coworkers discussed the applications of grafted chitosan in dye removal [68]. Gautam and coworkers prepared a Fe_3_O_4_ loaded chitin nanomaterial (MCH NM) adsorbent and tested the removal of Reactive Blue 13 (RB13) dye from aqueous solution [69]. Batch adsorption revealed adsorption activity of 199.02 mg/g towards RB13 dye. Kim et al. tested methylene blue adsorption properties of poly(vinyl alcohol) (PVA) nanofibrous membranes (NFMs) fabricated with chitin nanowhiskers (CtNWs) or chitosan nanowhiskers (CsNWs) [70]. The xanthated chitosan/cellulose sponges prepared by Xu and coworkers displayed maximum adsorption capacities of 213.220 and 289.855 mg/g toward methylene blue (MB) and Congo red (CR), respectively [71]. Ionic liquid (1-butyl-3-methylimidazolium bromide (BmImBr)) impregnated chitosan beads were prepared for removal of methylene blue by Karimi-Maleh et al. [72]. The maximum MB adsorption capacity of 188.68 mg/g was reported for chitosan-nickel oxide (CS-NiO) [73]. Ramakrishnan et al. prepared a lightweight Karaya gum (Kg) and chitosan (Ch) conjugate sponge (Kg-Ch sponge), which exhibited adsorption capacities of 32.81 and 32.62 mg/g for anionic dye methyl orange (MO) and cationic dye methylene blue (MB) [74]. Morais da Silva and coworkers developed low-cost chitosan-based beads for basic blue 7 (BB7) dye uptake using both batch and fixed-bed column adsorption [75]. The best BB7 adsorption capacity was 1410 mg/g. They also showed a simultaneous adsorption process of BB7 in the presence of another dye, basic brown 4 (BB4). The adsorption capacities of 232 and 259 mg/g were observed for basic brown 4 (BB4) and BB7 during simultaneous adsorption process. CuO oxide nanowires incorporated chitosan (CS) and polyvinyl alcohol (PVA) polymeric nanocomposite (CS-PVA@CuO) were used for the removal of Acid Blue 25 (AB25) with an adsorption capacity of 171.4 mg/g [76]. Table 2 summarizes some of the recent dye removal studies carried out using cellulose, chitin and chitosan-derived adsorbents [25,26,58,59,60,61,62,63,64,65,66,67,68,69,70,71,72,73,74,75,76,77,78]. Generally, the adsorption capacity of an adsorbent is governed by the source and the surface properties such as porosity and surface area. Among those many adsorbents listed in Table 2, adsorbents with higher surface properties such as higher micropore volume, smaller pore size and higher surface area showed higher adsorption capacities. Therefore, cellulose-based carbon aerogels exhibited the highest adsorption capacity of 1947 mg/g against MG mainly due to their improved surface properties, including reduced micropore diameter, increased micropore volume and relatively large surface area [60]. While adsorption technologies are widely studied for dye removal, they have several drawbacks as well. The dye adsorption mechanism of cellulose, chitin and chitosan adsorbents is mainly dependent on the type of dye. For instance, dyes (cationic, anionic, amphiphilic, and neutral) predominantly adsorb via dipole–dipole, hydrogen bonding or electrostatic interactions. However, the carbonaceous materials, including biopolymer-derived activated carbon, interact via dipole–dipole, hydrogen bonding, electrostatic, π-π and hydrophobic interactions. Scheme 1 depicts the three major adsorption mechanisms of dye molecules (cationic MB and anionic MO) by cellulose, chitin, chitosan and carbon materials. Table 3 summarizes the main advantages and disadvantages of the adsorption processes currently applied in the industry.

### 3.2. Advanced Oxidation Processes (AOPs)

Recently, advanced oxidation processes (AOPs) are considered one of the most attractive and effective dye removal technologies, broadly for the treatment of groundwater and surface water soil remediation. This AOP technology was first proposed by William Glaze and company in 1987 [77,79]. AOPs are based on the in situ generations of strong oxidants including peroxides (O_2_^2–^), superoxide (O_2_^•−^), hydroxyl radicals (OH^•^) and sulphate radicals (SO_4_^•–^) for chemical oxidation of recalcitrant organic compounds and removal of certain inorganic pollutants [25,26]. This process is also known as in situ chemical oxidation. During the oxidation process, those reactive species engage in complete degradation of large and complex organic compounds, including industrial dyes into intermediates and subsequently mineralizing those intermediates into water and simple inorganic compounds and ions such as carbon dioxide (CO_2_), sulfates (SO_4_^2–^), chloride (Cl^–^) and nitrates (NO^3–^), without being transferred into another phase [68,69]. The key advantages and disadvantages of AOPs are summarized in Table 4 [25].

AOP technologies mainly involve photocatalytic processes, Fenton-like processes, ozonation processes, semiconductor photocatalysis, catalytic oxidation (non-iron), ultrasound irradiation, electronic beam irradiation or a combination of two or more processes. Examples of most widely used AOPs include photocatalytic oxidation (UV, UV/H_2_O_2_, UV/Fe_2_^+^, UV/H_2_O_2_/Fe_2_^+^, UV/O_3_, UV/S_2_O_8_^2−^, UV/Cl_2_), ozonation (O_3_, O_3_/ultraviolet (UV), O_3_/H_2_O_2_, O_3_/H_2_O_2_/UV), semiconductor photocatalytic oxidation (UV/TiO_2_, UV/ZnO, UV/SnO_2_, UV/CeO_2_, UV/ Bi_2_O_3_, UV/WO_3_, UV/NiO, UV/CuO), catalytic homogeneous oxidation (oxides of Mn, Cu, Ru, Ag, and Co), catalytic heterogeneous oxidation (NiO/Al_2_O_3_, Cu/Li_2_O/γ-Al_2_O_3_), colloidal metal nanoparticles (Au, Ag, and Pd), ultrasound irradiation, Fenton reactions (Fe_2_^+^/H_2_O_2_, Fe_2_^+^/H_2_O_2_/UV, Fe_3_^+^/H_2_O_2_/UV) and electrochemistry (anodic oxidation and electro-Fenton) [26,79,80,81,82,83].

The role of biopolymers in the AOPs has gained significant attention over the last few years. Biopolymers including cellulose, chitin and chitosan have been widely utilized as solid substrates for AOPs. Biopolymers have tremendous benefits as a substrate due to their relative abundance, ease of surface functionalization, synergetic effects, and flexibility with varying degrees of crystallinity [84]. The presence of surface hydroxyl groups also makes biopolymers strong reducing and stabilizing agents [84]. Moreover, biopolymers can also prevent self-aggregation of nanoparticles preserving their shape and morphology. This section aimed to review recent examples of using three main biopolymer-based substrates, including cellulose, chitin and chitosan in the semiconductor photocatalytic and catalytic oxidation processes for dye removal.

Rajagopal et al. prepared banana pseudostem-derived micro-cellulose (MC) and titanium dioxide (TiO_2_) composites (TiO_2_+MC) and investigated the photocatalytic degradation of cationic (methylene blue and methyl violet) and anionic (acid violet) dyes [85]. They also found an enhancement of dye degradation with the addition of H_2_O_2_. The best degradation efficiency of 99% was observed for MB in 150 min. Complete degradation of AV and MV was achieved in 6 and 7 h reaction time, respectively. The reusability of the material was also tested for four consecutive cycles. Cellulose acetate-polyurethane (CA-PU) membrane impregnated with nano ZnO as a photocatalyst was prepared by Rajeswari et al. and tested for the removal of reactive red (RR 11) and reactive orange (RO 84) using UV-light under different experimental conditions. They reported a maximum degradation of dyes in 40 min at pH 7. The second-order rate constants obtained for RR 11 and RO 84 were 19.9123 and 13.2749 mg/L/min [86]. Thomas and coworkers studied the photocatalytic degradation of Congo red dye (CR) using titanium dioxide (TiO_2_)-nanoparticles (NPs) encapsulated alginate (Alg)-carboxymethyl cellulose (CMC) nanocomposite hydrogels crosslinked with barium (Ba) ions [65]. Ba/Alg/CMC/TiO_2_ composite hydrogels exhibited over 90% photocatalytic activity in 4 h towards Congo red dye under direct solar light irradiation. The first-order rate constant for the photodegradation of MB by Ba/Alg/CMC/TiO_2_ composite hydrogels was found to be 7.96 × 10^−3^ min^−1^. The authors also reported photocatalytic degradation of over 85% up to four consecutive cycles [87].

Ren et al. examined zeolitic imidazole framework (ZIF)/cellulose hybrid aerogels for activating peroxymonosulfate (PMS) to degrade organic pollutants, including rhodamine B (RB), tetracycline hydrochloride (TC) and p-nitrophenol (PNP) [66]. PMS was effectively activated by hybrid aerogels to produce SO_4_^•–^ and ^•^OH. The hybrid aerogels/PMS system could degrade the rhodamine B (RB) about 99% in 10 min [88]. Zhu and coworkers fabricated α-Fe_2_O_3_ nanodisk/bacterial cellulose hybrid membranes and tested the degradation of various cationic dyes (Rhodamine B (RhB), methylene blue (MB), crystal violet (CV) and malachite green (MG)) and anionic dyes (methyl orange (MO), orange ΙΙ (OII)) using sulfate radicals generated in a continuous flowing bed device [89]. Peroxymonosulfate (PMS) was used as a sulfate radical source produced under visible light. They showed the degradation of both cationic and anionic organic dyes under a flowing bed state for at least 84 h with a catalytic efficiency of up to 100% under optimized hybrid conditions. The cationic dye, RhB, was photodegraded to 100%, 93% and 87% upon irradiation for at least over 84 h (flow rate, 3 mL/h), 62 h (flow rate, 6 mL/h) and 42 h (flow rate, 9 mL/h), respectively. The anionic OII was effectively photodegraded over 94% in the flow range of 8–16 mL/h [82]. Diao et al. prepared cellulose nanocrystals (CNC)/manganese dioxide (MnO_2_)/alginate (ALG) beads and tested them for the adsorptive and oxidative degradation of methylene blue dye [90]. CNC/MnO_2_/ALG beads exhibited decolorizing efficiency and adsorption capacity of 99.8% and 136.7 mg/g, respectively. Their material also demonstrated high decolorizing efficiency of over 95% even after 10 cycles [90].

Dassanayake and coworkers investigated the adsorptive and photocatalytic degradation of methylene blue (MB) dye using an aerochitin–anatase TiO_2_ composite [77]. They also reported dual function of chitin aerogels (or aerochitin) as a sorbent and a supporting matrix enhancing the photocatalytic degradation properties of TiO_2_. Aerochitin–TiO_2_ composite showed excellent adsorptive and photocatalytic activity with a degradation degree of 98% for MB. The first-order rate constants for the photodegradation of MB by TiO_2_ nanoparticles and aerochitin–TiO_2_ composite were found to be (3.49 ± 0.04) × 10^−3^ and (1.82 ± 0.02) × 10^−2^/min [77]. They also proposed a degradation pathway of the dye using mass spectrometric analysis of the degradation products [77]. The hybrid material prepared from chitin and MnO_2_ was used to effectively remove MB [91]. Their MnO_2_–chitin hybrid material exhibited a complete oxidative decolorization of MB in 2.5 min with degradation efficiency of 99% over ten consecutive cycles [91]. Drumm et al. prepared an iron oxide impregnated mesostructured chitin template, ZSM-5, and tested for its application as a photo-Fenton catalyst to remove tartrazine dye under visible light irradiation [92]. Their results demonstrated a 95% decolorization of the dye at 30 min of reaction, and significant mineralization of 80% was observed at 180 min. A novel chitin graphene hydrogel (CGH) supported zinc oxide-graphene oxide (ZnO–GO) composite was investigated by Di and coworkers for the removal of methylene blue (MB) under visible light and showed a photocatalytic performance of 99% after 150 min [93]. Their recyclability studies also showed a photodegradation efficiency of 93% after five repetitions.

The self-assembled gel of iron (Fe)-chitosan (CS)/montmorillonite nanosheets (FeCS/MMTNS) was prepared by Zhao et al. for the degradation of methylene blue (MB) under visible light in the presence of H_2_O_2_ [94]. The kinetic rate constant of MB degradation on the FeCS/MMTNS substrate was 0.2261 mg/L/min. A complete decolorization of MB was observed in 120 min due to the synergetic absorption and H_2_O_2_ catalyzed photo-Fenton reaction [94]. The cycling experiments for the MB degradation with Fe-CS/MMTNS showed high efficiency of over 90% even after five cycles [94]. Aziz et al. developed chitosan zinc sulfide nanoparticles (CS-ZnS-NPs) as an efficient photocatalyst for the degradation of two carcinogenic azo dyes, Acid Brown 98 and Acid Black 234 [95]. They used a UV lamp (254 nm) as an irradiation source during the photocatalysis process. Synthesized CS-ZnS-NPs showed 96.7% degradation for Acid Black 234 in 100 min and 92.6% for Acid Brown 98 in 165 min. The pseudo-first-order rate constants of the degradation were 0.01464 and 0.04096/min for Acid Brown 98 and Acid Black 234. The CS-ZnS-NPs were easily recovered and recycled for four successive batches with 85% and 89% photocatalytic efficiency for Acid Brown 98 and Acid Black 234 [95].

Two types of chitosan-based composites (chitosan (CH)/ZnO and chitosan (CH)/Ce–ZnO composites) were synthesized under microwave irradiation and applied in photocatalytic removal of malachite green (MG) dye under a visible light source [96]. Complete photocatalytic removal of 5 mg/L MG was observed after 90 min and 60 min with 0.05 g of CH/ZnO and 0.03 g of CH/Ce–ZnO, respectively. Reusability tests showed an MG degradation efficiency of 84% and 90% for CH/ZnO and CH/Ce–ZnO after five cycles of reusing. Visible-light-driven ternary metal (Iron, nickel, and selenide) nanoparticles (FeNiSe-NPs) incorporated by chitosan (CHM) microspheres (FeNiSe-CHM) were prepared by Yang and coworkers and investigated for the photocatalytic degradation of Congo red (CR) dye [97]. The catalyst microspheres displayed photocatalytic degradation efficiency of up to 99% for CR under optimized conditions of 140 min, pH 6.0, dye concentration of 60 ppm and catalyst dose of 0.2 g. After five consecutive cycles, FeNiSe-CHM showed a degradation efficiency of over 90% [97]. Vigneshwaran and coworkers synthesized an integrated, flexible TiO_2_ imprinted chitosan/hydroxyapatite (TiO_2_@CS-Hpt) composite and investigated the degradation of Methylene Blue (MB) and Rhodamine B (RhB) dyes via photo-degradation method [98]. They observed a maximum degradation efficiency of 98.6% and 96.7% under 120 min of light exposure for MB and RhB, respectively. The authors also reported photocatalyst reusability up to seven consecutive cycles [98].

**Scheme 2:**
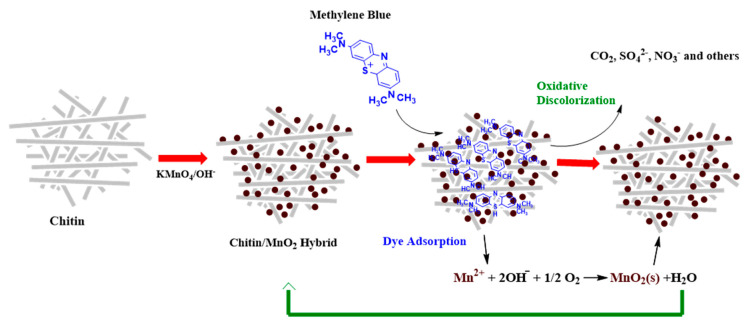
Schematic representation for the possible mechanism of decolorization of MB on MnO_2_–chitin hybrid. (Reprinted with permission from reference [91], Copyright 2016 Elsevier).

During the removal of dyes by AOPs, biopolymers do not directly involve the oxidative degradation mechanisms. However, biopolymers play a significant role as a matrix anchoring the oxidants. Biopolymer matrices exhibit enhanced surface properties, including high surface area and micropore volumes, providing more active sites for dye adsorption. The adsorption of dyes onto the catalytic surface is a prerequisite to the efficient oxidation of dyes [91]. Scheme 2 displays the schematic representation of the mechanism for the oxidative decolorization of MB on the MnO_2_–chitin hybrid proposed by Dassanayake et al. [90]. As seen, chitin acts as a substrate for both the catalyst and the dye, increasing the interaction between the dye and the catalyst. The authors reported that the high oxidative decolorization efficiency of MB is due to increased dye adsorption by chitin [91]. Table 5 compares dye degradation efficiencies of the biopolymer-based AOP processes discussed in this review [78,86,87,88,89,90,91,92,93,94,95,96,97,98,99].

### 3.3. Membrane Filtration

Membrane filtration is an advanced treatment technology and has exhibited significant potential in dye removal applications. Membrane filtration possesses intriguing characteristics, including high efficiency, sustainable operation, and low cost [99]. This technique involves passing the wastewater through membranes with small pores, and the membrane acts as a selective barrier trapping the solutes larger than the pore size [4]. The solution that passes through the membrane is free from solutes. The solutes retained on the membrane form a layer of filter cake and must be cleaned regularly to maintain a smooth running of the filtration process [34]. Wastewater that passes through the membrane is called the permeate, and the water and solutes that are rejected by the membrane are called the concentrate or reject [100]. Most commonly used membrane filtration techniques include low-pressure membrane processes including microfiltration (MF), ultrafiltration (UF) and nanofiltration (NF) and high pressure-driven processes such as reverse osmosis (RO) [4]. The pressure gradient across the membrane is referred to as the transmembrane pressure (TMP) and acts as the driving force for water migration [100]. Pressure-driven membrane filtration is based on the sieving effect and the physical or chemical interactions of separated components with the membrane [101]. The sieving effect is the separation of rejected solutes, including ions, molecules, colloid particles or microparticles, according to the size of pores on the membrane [101]. This effect is commonly applied in MF and UF techniques. Physical interactions, including electrostatic repulsions between charged solutes such as divalent ions, charged colloids and amino acids with the charged membrane, are involved in the separations by NF or UF techniques [101]. The separation of solutes using chemical interactions includes the formation of complexes with solutes and catalytic splitting of solutes [101]. The separation process in the RO technique is based on differences in sorption or solubility of the solutes during the solution-diffusion process. Table 6 summarizes the types of different membranes and their features. Like any other dye removal technique, membrane filtration also possesses advantages and limitations, see Table 7.

The efficiency of the membrane relies on its pore size, mechanical strength, surface charge and resistance to cleaning chemicals [100]. Commonly used pressure-driven membranes are made of synthetic organic polymers, including polysulfone, polyethersulfone, polypropylene (PP), polytetrafluorethylene (PTFE) and polyethylene (PE) [103,104,105]. However, membrane fouling is a critical issue in all pressure-driven membranes reducing the flux and life span of the membrane. The membrane fouling is caused by the deposition of particles, molecules or colloids onto the membrane surface by chemical, physical, mechanical actions and subsequently blocking the membrane pores. Biopolymers have been recently considered as antifouling agents in membrane separation applications due to hydroxyl groups and easy surface functionalization. These antifouling agents improve the surface morphology, charge and hydrophilicity of the membrane, enhancing productivity and life span. For instance, Li et al. reported the fabrication of permeable and antifouling UF membranes using bacterial cellulose with mussel-inspired dopamine and graphene oxide [106]. Graphene-oxide nanosheet functionalized phosphorylated chitosan (PCS) NF membranes also exhibited high flux, separation efficiency and antifouling performance [107]. Therefore, due to their unique characteristics such as high mechanical strength, tunable weight, surface properties, strong networking abilities and antifouling, biopolymers and their derivatives have been used to devise pressure-driven membranes.

Song and coworkers prepared a three-dimensional (3D) nanofibrous UF membrane using TEMPO (2,2,6,6-tetramethylpiperidine-1-oxylradical) oxidized cellulose nanofibers (TOCN) and zeolitic imidazolate frameworks (ZIF-8) [104]. The thickness of the UF membrane was 20 μm with 84 L/m^2^/h/bar water flux after 24 h filtering, displaying high durability against applied pressure in the range of 1–3 bar. Cellulose-based UF membrane showed excellent selective removal of cationic dye, Janus Green B (JG B), in the presence of anionic dyes due to strong electrostatic interaction via negatively charged TOCN networks. The rejection rate of the cationic dye was 99% from the dye mixture. Weng et al. reported the preparation of bamboo cellulose-based nanofiltration membrane (BC-NFM) and evaluated the retention rate and water flux of nanofiltration of methyl orange (MO) and methyl blue (MB) dyes [108]. At 0.5 MPa applied pressure, the retention rate to MO and MB were 93.0% and 98.9%, and the water flux was 12.31 L/m^2^/h and 10.12 L/m^2^/h, respectively [108].

A nanofiltration membrane was prepared using cellulose hollow fibers via a spinning technique using three different ionic liquids as solvent by Falca et al. [109]. The hollow fiber membrane performance was investigated using different charged dyes in ethanol and water. However, the negatively charged Congo red dye showed a rejection rate >90% in ethanol and approximately 100% in water with the highest water flux of 48 L/m^2^/h/ bar [109]. Bai and coworkers prepared cellulose nanocrystals incorporated polyamide (PA) thin film composite (TFC) nanofiltration membranes (CNC-TFC-Ms) for desalination and dye removal applications [110]. The CNC-TFC-Ms showed great removal performance for both anionic (rose Bengal (RB), Congo red (CR) and methyl orange (MO)) and cationic dyes (crystal violet (CV) and methylene blue (MB)) over 99.0% rejection efficiency.

Fradj et al. reported the fabrication of polymer enhanced ultrafiltration (PEUF) cellulose membrane using chitosan (CHI) as chelating polymer and its ability to separate azoic dyes (methyl orange (MO) and Direct Blue 71 (DB71)) from their aqueous solutions against applied pressure in the range of 0–4 bar [111]. CHI enhanced ultrafiltration showed retention rate and permeate flux of 86%, 37. 85 L/h/m^2^ and 89%, 25. 72 L/h/m^2^ for methyl orange (MO) and Direct Blue 71 (DB71), respectively [111]. They observed an increase in dye retention at the pH range from 2 to 6 and a decrease in dye retention with increasing ionic strength. Mokhena and coworkers fabricated three-tier composite membranes composed of alginate nanofibers, electrospun onto a mechanical support layer of non-woven polyethylene terephthalate (PET) fabric as a mid-layer, and coated with silver nanoparticles containing chitosan (CaA-AgNPs) as a selective barrier layer [112]. The authors also used calcium and glutaraldehyde as dual crosslinking agent to improve the stability of electrospun alginate nanofibers. They showed 95% rejection efficiency of Congo Red (CR) dye for silver nanoparticles containing chitosan (CaA-AgNPs) membrane over five filtration cycles. Table 8 summarizes the dye rejection efficiencies of biopolymer-based membrane filtration technologies discussed above.

## 4. Conclusions

Dye effluents are one of the major causes of pollution of water bodies and have profound negative implications both from an environmental and health perspective. Technologies including biological, chemical, and physical have been employed for dye removal. However, many dye removal technologies are not energy efficient and cost-effective, making them less popular. Biopolymers including cellulose, chitin and chitosan are known for their relative abundance, renewability, non-toxicity, biodegradability and ease of functional modifications. Therefore, they are widely considered for the removal of dyes from wastewater. In this paper, recent developments on the use of cellulose, chitin and chitosan in the three most important biopolymer-based technologies, namely, adsorption, advanced oxidation processes and membrane filtration, were extensively discussed. Notably, the role of biopolymers in dye removal technologies has been marked as promising and cost-effective compared to currently available techniques.

## Figures and Tables

**Table 1:** Different types of dyes and their main applications. (Adapted from reference [8], Copyright 2018 Elsevier).

Type	Water Solubility	Applications	Common Application Method	Example
Acid	Soluble	Cosmetics, food, leather, modified acrylics, nylon, paper, printing ink, silk and wool	In dye baths with neutral to acidic conditions	Acid Yellow 36
Azo	Soluble/insoluble	Acetate, cellulose, cotton, rayon and polyester	Coupling component used to impregnate fiber and a solution of stabilized diazonium salt is used for treatment	Bluish Red azo dye
Basic	Soluble	Inks, medicine, modified nylon, modified polyester, paper, polyacrylonitrile, polyester, silk, tannin, mordanted cotton and wool	In dye baths with acidic conditions	Methylene Blue
Direct	Soluble	Cotton, leather, nylon, rayon, silk and paper	In dye baths with neutral or slightly alkaline conditions with additional electrolyte.	Direct Orange 26
Disperse	Insoluble	Acetate, acrylic fibers, cellulose, cellulose acetate, nylon, polyamide, polyester, polyester–cotton and plastic	Padded on cloth and either baked or thermo-fixed at high pressure and temperature or low temperature carrier methods	Disperse Blue 27, Disperse Red 4, Disperse Yellow 3
Fluorescent brighteners		All fibers, oils, paints, plastics and soaps as well as detergents	Mass dispersion, solution or suspension	4,4′-bis (ethoxycarbonylvinyl) stilbene
Food, drug, and cosmetics		Food, drug, and cosmetics	Mixing	Food Yellow 4 and tartrazine
Mordant		Anodized aluminum, natural fibers, leather and wool	Along with chromium salts	Mordant Red 11
Oxidation bases		Cotton, fur and hair	The substrate is oxidized with aromatic amines and phenols	Direct Blue
Reactive	Soluble	Cellulosic, cotton, nylon, silk and wool	Reaction between functional group on fiber and reactive group on dye. Covalently bonding under heat and alkaline pH	Reactive Blue 5
Solvent	Insoluble	Fats, gasoline, inks, lacquers, lubricants, oils, plastics, stains, varnishes and waxes	Substrate dissolution	Solvent Red 26, Solvent Blue 35
Sulfur		Cotton, leather, paper, polyamide fibers, rayon, silk and wood	Aromatic substrate vatted with sodium sulfide and reoxidized to insoluble sulfur-containing products on fiber	Sulfur Black 1
Vat	Insoluble	Cotton, cellulosic, polyester–cotton, rayon and wool	Water-insoluble dyes solubilized by reducing with sodium hydrogen sulfite, then exhausted on fiber and reoxidized	Vat Blue 4 (Indathrene)

**Table 2:** Adsorption of industrial dyes using biopolymer-based adsorbents.

Adsorbent (s)	Dye(s)	Maximum Adsorption Capacity, Qm (mg/g)	Ref.
Cellulose-based adsorbent derived from sugarcane bagasse	PB	9.6	[56]
Carboxymethyl cellulose-derived activated Carbon	MV AR CR	1351.4 223.2 1779.5	[57]
N-doped carboxymethyl cellulose-based carbon aerogels	MG MB CR TRB G133	238.2 230.4 85.2 73.3	[58]
Cellulose-based carbon aerogels	MG MB	1947 1192	[59]
Activated carbon-containing cellulose beads	TB	123.5	[60]
3D cellulose-based microspheres	AO7	218.6	[61]
Cellulose nanofibril aerogels	MG	212.7	[62]
MCMFCs composite beads	MB	303	[64]
CNC/PMVEMA/PEG	MB	116.2	[65]
Fe3O4 loaded MCH NM	RB13	199.02	[68]
Xanthated chitosan/cellulose sponges	MB CR	213.220 289.855	[70]
CS-NiO	CR	188.68	[72]
Kg-Ch sponge	MO MB	32.81 32.62	[73]
Chitosan-based beads	BB7	1410	[74]
CS-PVA@CuO	AB 25	171.4	[75]

**Table 3:** Advantages and disadvantages of the adsorption techniques.

Advantages	Disadvantages
Technologically simple, easy adaptability and handling	Economically non-viable due to the relatively high cost of adsorbents
High dye adsorption	Non-selective method and non-efficient with certain types of dyestuff
Highly effective process with fast adsorption kinetics	Surface modification is required to improve their adsorption capacity on certain dyes
Excellent ability to remove a wide range of dyes and other industrial effluents	Adsorption efficiency depends on the type of material
Non-destructive process	Elimination of the adsorbates requires generation, replenishment or incineration of the adsorbent
Produce high quality treated effluents	Regeneration of the adsorbent is difficult and expensive

**Table 4:** Key advantages and disadvantages of AOPs used for dye removal.

Advantages	Disadvantages
No pH adjustments	High chemical and energy consumption
High discoloration efficiency	Low performances for COD removal
Biodegradability improvement	UV radiation requirement for some AOPs
Toxicity reduction	Technology complexity
Free of sludge production	Inorganic sludge production

**Table 5:** Comparison of dye degradation efficiencies of the biopolymer-based AOP processes.

Catalyst (s)	Dye(s)	Degradation Efficiency, %	Time, min	Reusability, #of Cycles	Ref.
TiO_2_+MC composites	MB MV AV	99 100 100	150 420 360	4	[84]
CA-PU-ZnO membrane	RR 11 RO 84	<95 <90	40	-	[85]
Ba/Alg/CMC/TiO_2_	CR	<90	240	4	[86]
ZIF/cellulose aerogels	RhB	99	10	5	[87]
α-Fe_2_O_3_ nanodisk/bacterial cellulose membranes	RhB, MB, CV, MG, MO, OII	100	5040	-	[88]
CNC/MnO_2_/ALG	MB	99.8	5	10	[89]
Aerochitin–TiO_2_ composite	MB	98	200	-	[76]
Chitin-MnO_2_	MB	99	2.5	10	[90]
ZSM-5	Tartrazine dye	95	30	-	[91]
Chitin graphene hydrogel (CGH) supported ZnO–GO	MB	99	150	5	[92]
FeCS/MMTNS	MB	100	120	5	[93]
CS-ZnS-NPs	AB 98 AB 234	92.6 96.7	165 100	4	[94]
CH/ZnO CH/Ce–ZnO	MG	100	90 60	5	[95]
FeNiSe-CHM	CR	99	140	5	[96]
TiO_2_@CS-Hpt	RhB MB	96.7 98.6	120	7	[97]

**Table 6:** Key features of pressure-driven membrane filtration processes. Adapted from [100,102].

Membrane Process	Molecular Weight Cut-Off, kDa	Retained Diameters, μm	TMP Required, kPa	SystemRecovery
MF	100–500	10^−1^–10	10–100	90–99^+^
UF	20–150	10^−3^–1	50–300	85–95^+^
NF	2–20	10^−3^–10^−2^	200–1500	75–90^+^
RO	0.2–2	10^−4^–10^−3^	500–8000	60–90

**Table 7:** The advantages and limitations of membrane filtration technologies.

Advantages	Disadvantages
Technologically simple	Economically non-viable due to high operational and maintenance costs
Rapid and highly efficient	High energy requirements
Eliminates all types of dyestuffs	Membrane fouling
No chemicals used	Limited flow rate and low throughput
Non-destructive separation technique	Sludge formation and high cleaning and membrane regeneration costs
A wide range of membranes available	Choice membrane system depends on the type of the dye

**Table 8:** Dye rejection efficiencies of the biopolymer-based membrane filtration technologies.

Membrane	Membrane Pressure, kPa	Dye(s)	Rejection Efficiency, %	Permeate Flux, L/h/m^2^	Ref.
UF/TOCN/(ZIF-8)	100–300	JG B	99	84	[103]
BC-NFM	500	MO MB	93.0 98.9	12.31 10.12	[107]
NF cellulose hollow fibers	20	CR	100	48	[108]
NF- CNC-TFC-Ms	60	RB CR MO CV MB	99	-	[109]
PEUF/cellulose/chitosan (CHI)	200	MO DB71	86 89	37.85 25.72	[110]
PET-CaA-AgNPs	2.07	CR	95	-	[111]

## Data Availability

Not applicable.

## Sample Availability

Samples of the compounds are not available from the authors.
